# Effects of L-Citrulline Supplementation on Rumen Microbiota and Reproductive Performance of Ewes

**DOI:** 10.3390/life16050766

**Published:** 2026-05-02

**Authors:** Tingting Lu, Hui Chen, Jiaqi Liu, Tingting Li, Hao Lu, Reylağül Rehim, Haibo Lv, Chenyang Gao, Guodong Zhao, Kailun Yang

**Affiliations:** 1 College of Animal Science, Xinjiang Agricultural University, Urumqi 830052, China; lutt11071024@163.com (T.L.);; 2 Huishang Ecological Animal Husbandry Co., Ltd., Toksun County, Turpan 838100, China

**Keywords:** L-Cit, rumen microbiota, oestrus rate, conception rate, reproductive performance

## Abstract

This study aimed to investigate the effects of L-Citrulline (L-Cit) supplementation on the rumen microbiota and reproductive performance of Turpan black ewes (each ewe was supplemented with 12 g of L-Cit daily). A total of 120 multiparous Turpan black ewes aged 1.5–2.5 years, with an average body weight of (52.35 ± 5.16) kg, were randomly divided into two groups: a control group (Con group) and an experimental group (L-Cit group), with 60 ewes in each group, for a 90-day supplementation trial. The results demonstrated that L-Cit supplementation regulated the rumen microbiota of ewes, increased the abundance of *Clostridia vadin BB60* in the rumen, and stimulated the synthesis and release of reproductive hormones. Blood samples for hormone analysis and rumen fluid for microbiota profiling were collected from a subset of six ewes per group (n = 6). Compared with the Con group, L-Cit supplementation numerically increased oestrus rate (93.33% vs. 77.55%, *p* = 0.32) but did not significantly affect reproductive hormone concentrations (*p* > 0.05 for all). Conception rate was 100% in both groups among bred ewes. No significant changes in rumen microbiota alpha diversity or community structure were observed.

## 1. Introduction

The reproductive capacity of ewes is determined by the success of oocyte development, fertilization, and embryo survival. Oocyte quality is of great importance for early embryonic development. L-Citrulline (L-Cit), a non-essential amino acid, is endogenously turned into L-arginine. So, it acts as an effective precursor for the creation of nitric oxide (NO) and polyamines, important signaling molecules involved in follicular growth, vascular function, and embryonic survival [1]. These metabolic pathways show that L-Cit supplementation may produce a positive effect on reproductive outcomes by adjusting systemic and local tissue physiological circumstances. Earlier work has found that including 10–15 g of L-Cit in the daily diet of each ewe can improve the plasma reproductive hormone levels, antioxidant status, and reproductive performance in Hu sheep [2,3]. Alike positive outcomes have been seen in rams, for example, better hormone profiles and antioxidant capacity [4,5].

As well as having systemic effects, L-Cit may modify the gastrointestinal microbiota. Although there is little direct evidence from ruminants, non-ruminant studies have shown that L-Cit and its metabolite L-arginine can modify gut microbiota composition, barrier function, and immune responses [6,7]. As the rumen has a complex microbial ecosystem strongly associated with host metabolism and health, it is reasonable to believe that L-Cit supplementation might affect rumen microbial communities in ewes, perhaps leading to better reproductive performance. In ruminants, emerging evidence suggests a direct link between rumen microbiota and host reproduction. For example, in dairy cows, alterations in rumen bacterial composition have been associated with changes in circulating steroid hormones and fertility outcomes [8]. Similarly, in sheep, dietary manipulations that shift rumen fermentation patterns have been shown to affect plasma LH and estradiol levels [9]. These ruminant-specific studies provide a stronger foundation for our hypothesis than earlier cross-species work.

Actually, the specific effects of L-Cit on the rumen microbiota and reproductive performance of female Turpan black sheep have not been investigated in a comprehensive and well-organized manner. Hence, this study was planned to evaluate the result of dietary L-Cit supplementation on the rumen microbial composition and reproductive traits of Turpan black sheep ewes, providing a scientific basis for its application in ruminant production.

In spite of the increasing interest in L-Cit as a nutritional modulator, the biochemical and molecular mechanisms that this non-essential amino acid uses to upgrade microbial ecology and reproductive function are not well-known. L-Cit supplementation would modulate the rumen microbiota composition and its metabolites, thereby influencing the hypothalamic–pituitary–gonadal (HPG) axis and reproductive hormone secretion in ewes. More research has to be carried out to determine the routes by which L-Cit impacts rumen microflora and reproductive processes, specifically at the molecular level.

## 2. Materials and Methods

### 2.1. Ethic Statement

All animal care and handling procedures in this study were conducted under the guidance of the Care and Use of Laboratory Animals in China and were approved by (protocol number: 2020032 and 2020024) the Animal Care Committee of Xinjiang Agricultural University (Urumqi, Xinjiang, China).

### 2.2. Experimental Materials

L-Citrulline (L-Cit) was purchased from Shandong Pingju Biotechnology Co., Ltd. (China, Jining), with a purity of 99.1%, ash content of 0.05%, and nitrogen content of 24%. A centrifuge (Model 5424) was obtained from Eppendorf AG (Germany, Hamburg). Enzyme-linked immunosorbent assay (ELISA) kits for the determination of gonadotropin-releasing hormone (GnRH; Cat. No. ml410381), follicle-stimulating hormone (FSH; Cat. No. ml497803), luteinizing hormone (LH; Cat. No. ml440758), progesterone (P_4_; Cat. No. ml036688), and estradiol (E_2_; Cat. No. ml450381) in ewe plasma were all purchased from Shanghai Enzyme-linked Biotechnology Co., Ltd. (China, Shanghai).

### 2.3. Experimental Design and Grouping

The trial was conducted from June 2025 to September 2025 at Huishang Ecological Animal Husbandry Co., Ltd. (Coordinates 87°14′05″–89°11′08″ E, 41°21′14″–43°18′11″ N), with average outdoor and indoor temperatures of 35.5 °C and 38.8 °C, respectively, 90-day supplementary feeding trial.

A total of 120 multiparous Turpan black ewes aged 1.5~2.5 years, with an average body weight of (52.35 ± 5.16) kg. All ewes were healthy, non-pregnant, and free from reproductive system diseases. They underwent unified deworming and immunization, with similar body weights and consistent condition scores, were randomly divided into 2 groups: a control group (Con group) and a treatment group (L-Cit group), with 60 ewes per group, for a 90-day supplementation trial. All experimental ewes received the same diet composition and nutritional levels, housed in separate pens. All ewes were fed a basal diet with energy and protein levels meeting NRC standards. The Con group was fed the basal diet, while the L-Cit group received the basal diet supplemented with 12 g/d·head of L-Cit. All ewes were injected with 2 mg of PGF2α two days before the trial (to synchronize the oestrous cycle across all ewes, allowing a uniform starting point for the dietary treatment and subsequent hormone sampling). The formal feeding period was designated as trial day 0. On days 7 and 14, blood was collected from the jugular vein of 6 fixed 18-month-old ewes per group into heparin sodium anticoagulant tubes (plasma samples collected should be stored for no more than 3 months, green-capped tubes with lithium heparin are used to obtain plasma). Plasma was prepared by centrifugation at 2000× *g* for 15 min and stored at −20 °C for the determination of estradiol (E_2_), progesterone (P_4_), gonadotropin-releasing hormone (GnRH), follicle-stimulating hormone (FSH), and luteinizing hormone (LH) levels. Rumen fluid was collected 2 h after L-Cit supplementation on day 8 and preserved in liquid nitrogen for microbial analysis. All ewes stopped receiving L-Cit supplementation on day 15 and underwent a 7-day oestrus detection period. Before detection, 12 healthy, robust, disease-free Turpan black rams with strong libido were selected and fitted with marking harnesses. Six detection rams were introduced into each pen. Detection sessions were held daily from 09:30 to 10:30 and 19:30–20:30, continuing until day 21. Patience was maintained during detection, avoiding unnecessary movement, providing ample space for the rams, and preventing fights. Ewes accepting mounting were considered in oestrus. After detection, ewes in oestrus were identified by ear tag and transferred to an empty pen. Two breeding rams were used for natural mating with artificial assistance, and mating dates were recorded. Oestrus rate was calculated during this period. Pregnancy diagnosis was performed using B-ultrasound on day 66, and conception rate was calculated (abdominal examination using a portable B-mode ultrasound machine (probe frequency 5.0 MHz)).

**Figure 1:**
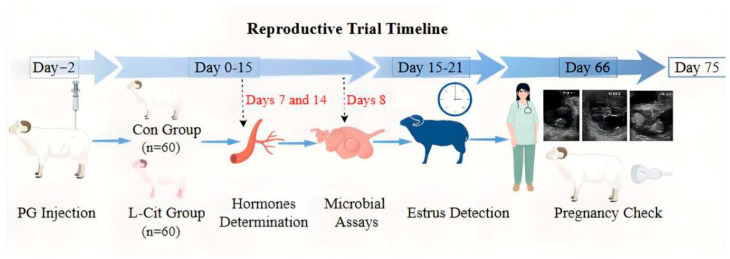
Experimental design diagram.

The L-Cit infusion and supplementation doses in this trial were based on studies by Gilbreath [10], Zhao [11], Ma [4], and An [12], who supplemented sheep with 10~12 g/d L-Cit. Based on the previous research by the scientific team, a daily dose of 12 g/d L-Cit was determined as the appropriate dosage under these experimental conditions. The specific experimental design flowchart is shown in Figure 1.

### 2.4. Animal Husbandry and Management

During the L-Cit supplementation trial, all multiparous Turpan black ewes were housed in groups in pens. There was feeding twice a day at 10:00 and 18:00. All of the sheep were allowed ad libitum access to feed and water.

Ewes were housed in a total of 4 pens, with 2 pens per treatment group and 30 ewes per pen (60 ewes per group; 120 ewes overall). The individual ewe served as the experimental unit for all measured variables. Feed intake was measured individually using Calan gates. Reproductive outcomes—including oestrus rate (proportion of ewes detected in oestrus), conception rate (proportion of bred ewes that became pregnant), and litter size—were recorded per ewe. Hormone concentrations and rumen microbiota composition were also analyzed at the individual ewe level (n = 6 per group for these subsets).

The daily dry matter intake was sort of 1.45 kg/d per ewe. Huishang Ecological Animal Husbandry Co., Ltd (Turpan, China) gave out the TMR mixed feed. Its composition and nutritional levels are shown in Table 1.

### 2.5. Sample Collection

#### 2.5.1. Plasma Sample Collection and Processing

On trial days 0, 7, and 14, before morning feed time, jugular venous blood of Turpan black ewes getting dietary L-Cit was collected. Blood was obtained and put into heparinized tubes. To get plasma, it was immediately spun at 2000× *g* for 15 min at 4 °C. Then, it was aliquoted into 2 mL cryovials and stored at −20 °C for the analysis of reproductive hormones later.

#### 2.5.2. Rumen Fluid Collection

2 h post-supplementation on day 8 of L-Cit feeding, rumen fluid (approximately 100 mL per ewes) was collected using a stomach tube-type rumen sampler, filtered through four layers of gauze, aliquoted into 5 mL cryovials, the rumen fluid was aliquoted into sterile cryogenic vials and immediately flash-frozen in liquid nitrogen to quench microbial metabolism and preserve nucleic acid integrity [13].

The particular way of collecting rumen fluid in this trial was:

(1) To reduce the effect of dietary rhythms on rumen microbial communities, rumen fluid was collected before morning feeding, making sure the experimental ewes were in a fasting state.
(2) For the purpose of collecting rumen fluid, a gastric tube-type sampler was used. It was made of a gastric tube, a vacuum bulb or pump, and a collection flask.
(3) Before it is used, the sampler should be fully assembled and have strict cleaning and disinfection done. One should rinse it with hot sterile distilled water and then purge it with anhydrous CO_2_ to expel air from the tubing, so as to set up an anaerobic environment.
(4) The gastric tube, lubricated beforehand, was slowly put through the mouth of the experimental ewe. Once the tube tip got to the dorsal or ventral part of the rumen, the vacuum bulb was linked.
(5) Dispose of the first 50 mL of rumen fluid and gather the subsequent rumen fluid into pre-warmed, sterile, anaerobic collection bottles. These bottles should have CO_2_ pre-filled and be maintained in a 39 °C water bath to keep the activity of rumen microorganisms.
(6) Move the rumen fluid that has been freshly collected to the laboratory at the earliest. With CO_2_ flowing continuously, pass the fluid through four layers of medical gauze to remove feed-related particles, protozoa, and impurities, getting a relatively pure rumen fluid filtrate.
(7) Hastily aliquot the filtered rumen fluid into pre-sterilized, 2 mL numbered cryovials. In order to prevent vial rupture due to volume expansion during freezing, fill the container to 80% of its capacity. This process should be carried out as speedily as it is possible under a continuous CO_2_ flow to keep anaerobic and low-temperature circumstances.
(8) Once aliquoting was done, the vials containing rumen fluid were put into liquid nitrogen to freeze rapidly. This makes sure the sample crosses the ice crystal formation zone within a very short period, maximizing the conservation of microbial RNA integrity or enzyme activity.
(9) After quick freezing of the samples, transfer them to an −80 °C ultra-low-temperature freezer for long-term storage until subsequent rumen microbial analysis.

The full collection and aliquoting process has to be strictly completed within 30 min to lessen oxygen exposure and its negative effects on anaerobic microorganisms.

### 2.6. Measurement Indicators

#### 2.6.1. Plasma Reproductive Hormones

Plasma levels of GnRH, FSH, LH, P_4_, and E_2_ were determined using enzyme-linked immunosorbent assay (ELISA) kits purchased from Shanghai Enzyme-linked Biotechnology Co., Ltd. (Shanghai, China) The specific methods can be referred to [14].

#### 2.6.2. Determination of Microorganisms in Rumen Fluid

DNA was purified from rumen fluid using the cetyltrimethylammonium bromide (CTAB) method (DNA extraction was performed exclusively from rumen fluid samples; fecal samples were not collected for microbial analysis) and used as a template for PCR amplification with specific primers. PCR products were quantified by fluorescence (Qubit 3.0). Total genomic DNA was extracted from rumen fluid samples using the QIAamp PowerFecal Pro DNA Kit. (Urumqi, China) The V3–V4 hypervariable region of the 16S rRNA gene was amplified using primers 338F (5′-ACTCCTACGGGAGGCAGCA-3′) and 806R (5′-GGACTACHVGGGTWTCTAAT-3′). Amplicon sequencing was performed on an Illumina MiSeq platform (2 × 300 bp paired-end). Raw reads were quality-filtered using Trimmomatic (v0.39), and chimeras were removed with VSEARCH (v2.15.0). Operational taxonomic units (OTUs) were clustered at 97% similarity using QIIME2 (v2021.8). Samples were sent to Shanghai Majorbio Bio-pharm Technology Co., Ltd. (Shanghai, China) for analysis. Specific methods can be referred to [15].

### 2.7. Statistical Analysis

Excel was used for preliminary organization of crude protein content, nitrogen metabolism indicators, and reproductive hormone data. For hormone concentrations measured repeatedly (days 0, 7, 14), used a linear mixed-effects model (SPSS 26.0) with treatment, time, and treatment × time as fixed effects, and ewes as a random effect. Pairwise comparisons were corrected using Bonferroni. Independent t-tests were retained for oestrus rate and conception rate comparisons. Oestrus detection began on day 15, designated as day 0 for oestrus rate statistics. Oestrus rate data were recorded at 0, 24, 48, 72, 96, 120, and 144 h over the 7-day detection period. oestrus rate and conception rate were analyzed using the Chi-square test in SPSS 26.0. The data underwent normality testing, and the differences between the two groups were compared using the independent samples t-test. The statistical power was set at 0.8, and the sample size was calculated based on the pre-test effect size.

Raw plasma metabolite data were processed using the metabolomics software Progenesis QI v3.0 (Waters Corporation, Milford, CT, USA) for peak extraction, alignment, identification, etc., resulting in a data matrix containing retention time, peak area, mass-to-charge ratio, and identification information for subsequent bioinformatics analysis, performed by Shanghai Majorbio Bio-pharm Technology Co., Ltd. (Shanghai, China).

Alpha diversity (Shannon, Chao1) was compared between groups using independent t-tests. Beta diversity (Bray–Curtis distances) was visualized by PCoA and tested by PERMANOVA (999 permutations). Differential abundant genera were identified using LEfSe (LDA threshold > 2.0) and DESeq2 (P-adjusted < 0.05). For both LEfSe and Spearman correlation analyses between genera and hormones, false discovery rate (FDR) correction was applied, with q < 0.05 considered significant.

Origin software (2025) was used to analyze correlations between reproductive hormones, gastrointestinal microbiota, and plasma metabolites, use Spearman correlation analysis and generate a heat map. Data visualization was performed using GraphPad Prism 9.0 and Adobe Illustrator 2025. Results are presented as mean ± standard error. *p* < 0.05 was considered statistically significant, and *p* < 0.01 was considered highly significant.

## 3. Results

### 3.1. Effects of L-Cit Supplementation on Ewes Reproductive Performance

The results in Table 2 showed that the oestrus rate of Con group was 61.67%, and that of L-Cit group was 73.33%. The conception rate in both groups was 100.00%. The oestrus rate of L-Cit group was 15.78% (*p* > 0.05) higher than that of Con group.

As the results show, a 7-day oestrus detection period began on day 15. In the Con group, oestrus rates were 5.00%, 11.67%, 23.33%, 33.33%, 46.67%, 56.67%, and 61.67% at 0, 24, 48, 72, 96, 120, and 144 h post-detection start, respectively, with the main oestrus period concentrated at 0~24 h, showing a significant increase of 133.40% (*p* < 0.05). In the L-Cit group, oestrus rates were 8.33%, 13.33%, 28.33%, 46.67%, 58.33%, 66.67%, and 73.33%, respectively, with the main oestrus period concentrated at 24~48 h, showing a significant increase of 240.10% (*p* < 0.05).

### 3.2. Effects of L-Cit Supplementation on Plasma Reproductive Hormone Levels in Ewes

**Figure 2:**
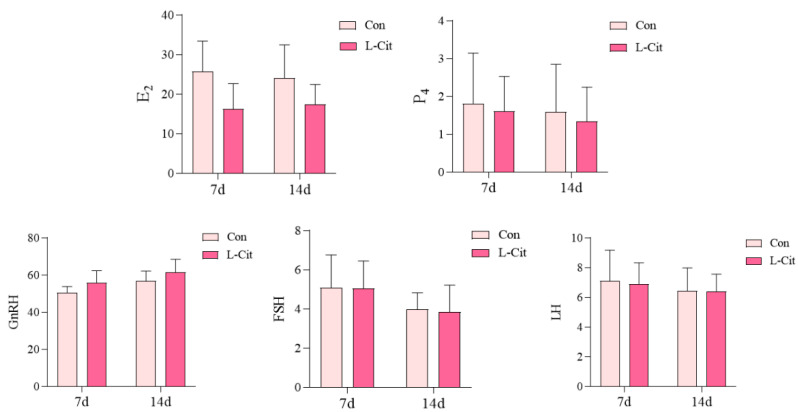
Effects of L-Cit supplementation on plasma reproductive hormone levels in ewes (n = 6). Note: Plasma concentrations of E_2_ (pg/mL), P_4_ (ng/mL), FSH (mIU/mL), and LH (mIU/mL) on days 0, 7, and 14.

As shown in Figure 2, on day 7 of the trial, the plasma GnRH levels in the L-Cit group increased by 11.17% compared to the Con group (*p* > 0.05), while the E_2_ levels decreased by 37.05% (*p* > 0.05), P_4_ levels decreased by 11.02% (*p* > 0.05), FSH levels decreased by 0.39% (*p* > 0.05), and LH levels decreased by 2.82% (*p* > 0.05). No significant differences were observed in hormone levels between the two groups. On day 14 of the trial, the plasma GnRH levels in the L-Cit group increased by 8.26% compared to the Con group (*p* > 0.05), while the E_2_ levels decreased by 27.56% (*p* > 0.05), P_4_ levels decreased by 15.14% (*p* > 0.05), FSH levels decreased by 3.76% (*p* > 0.05), and LH levels decreased by 0.08% (*p* > 0.05). No significant differences were observed in hormone levels between the two groups, with FSH and LH showing relatively small changes at all time points.

### 3.3. Effects of L-Cit Supplementation on Rumen Microbiota in Ewes

#### 3.3.1. Rumen Microbial Community Composition

**Figure 3:**
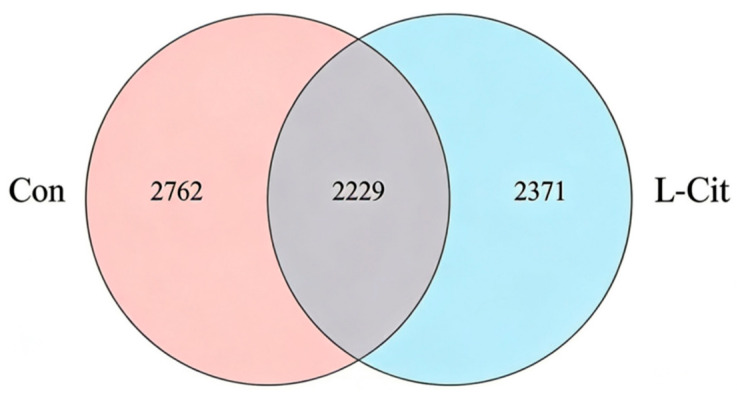
Venn diagram of species/operational taxonomic unit (OTU) analysis (n = 6).

After processing the sequencing data and clustering sequences at 97% similarity into OTUs, the results are shown in Figure 3. A total of 5133 different OTUs were identified between Con and L-Cit groups. Unique OTUs within Con and L-Cit groups were 2762 and 2371, respectively. The Con group had a total of 4991 OTUs, the L-Cit group had 4600 OTUs, and 2229 OTUs were shared.

#### 3.3.2. Alpha Diversity Analysis

**Figure 4:**
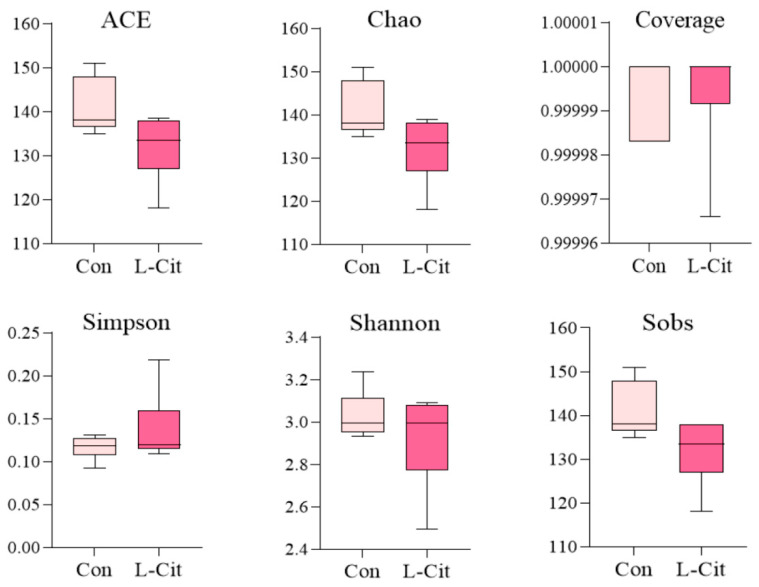
Box plots of alpha diversity indices (n = 6).

As shown in Figure 4, no significant differences were observed in the various indices between the Con group and the L-Cit group. Dietary supplementation of L-Cit did not significantly alter the diversity of the rumen microbial community in ewes. Furthermore, the species coverage for all samples reached 99.9%, indicating that the sequencing depth was sufficient to reliably represent the actual composition of the microbial community in the rumen samples.

#### 3.3.3. Principal Coordinates Analysis (PCoA)

**Figure 5:**
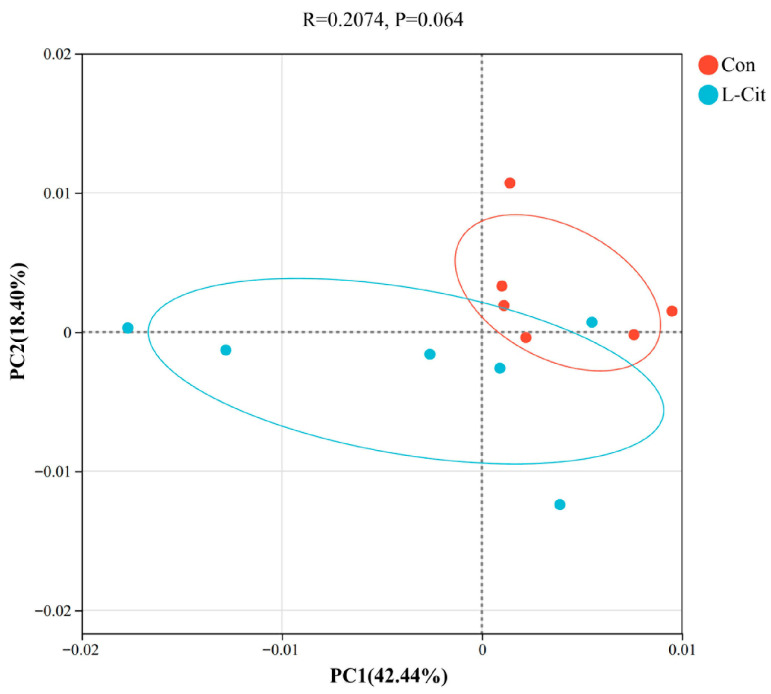
Principal Coordinates Analysis (PCoA) plot (n = 6). Note: The x-axis represents the first principal coordinate, and the y-axis represents the second principal coordinate. Scatter point shapes and colors denote different experimental groups.

As shown in Figure 5, the first principal coordinate explained 42.44% of sample variation, and the second explained 18.40%. Con and L-Cit groups were separated into two clusters. The Con group’s microbial composition was more compact with smaller community differences, while the L-Cit group’s composition was more dispersed with larger community differences.

#### 3.3.4. Effects of L-Cit Supplementation on Rumen Microbial Species Composition and Differential Impact

##### Effects at the Phylum Level

**Figure 6:**
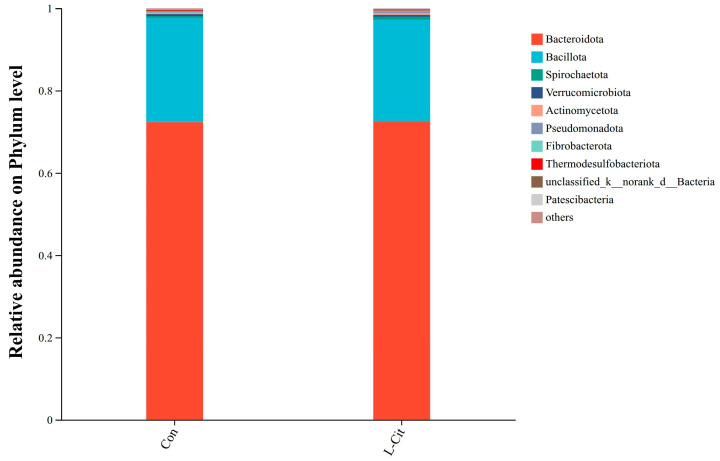
Relative abundance of rumen microbiota at the phylum level in ewes following dietary L-Cit supplementation (n = 6). Note: The x-axis represents the two treatment groups (Con = control; L-Cit = L-Citrulline supplemented). The y-axis shows the relative abundance (%) of each phylum. Different colored bars represent different phyla, and the bar length corresponds to the mean proportional abundance within each group. Error bars indicate standard deviation. Same below.

The composition of rumen microbiota in Con and L-Cit groups after L-Cit supplementation is shown in Figure 6. At the phylum level, major phyla included *Bacteroidota* (72.33%; 72.57%), *Bacillota* (25.33%; 24.62%), *Spirochaetota* (0.45%; 0.73%), *Verrucomicrobiota* (0.43%; 0.47%), and *Actinomycetota* (0.22%; 0.41%).

##### Effects at the Family Level

**Figure 7:**
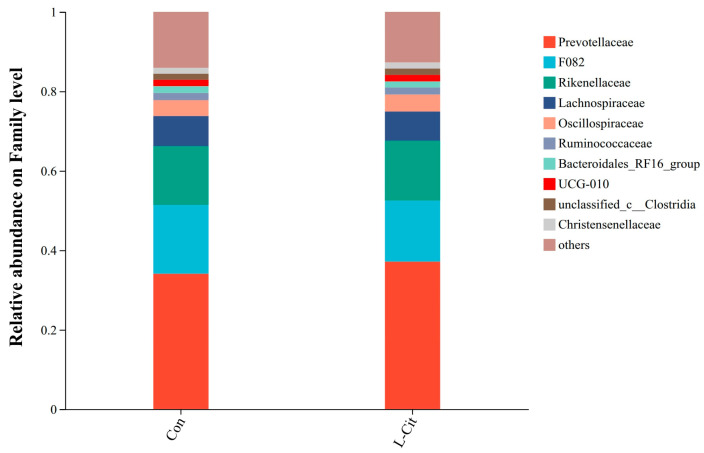
Relative abundance of rumen microbiota at the family levels in ewes following dietary L-Cit supplementation (n = 6).

As shown in Figure 7, at the family level, major families included *Prevotellaceae* (34.08%; 37.14%), *F082* (17.33%; 15.36%), *Rikenellaceae* (14.79%; 15.07%), *Lachnospiraceae* (7.59%; 7.34%), *Oscillospiraceae* (3.97%; 4.27%), *Ruminococcaceae* (1.83%; 1.78%), *Bacteroidales_RF16_group* (1.73%; 1.53%), *UCG-010*, *unclassified_c_Clostridia* (1.51%; 1.58%), and *Christensenellaceae* (1.52%; 1.57%).

##### Effects at the Genus Level

**Figure 8:**
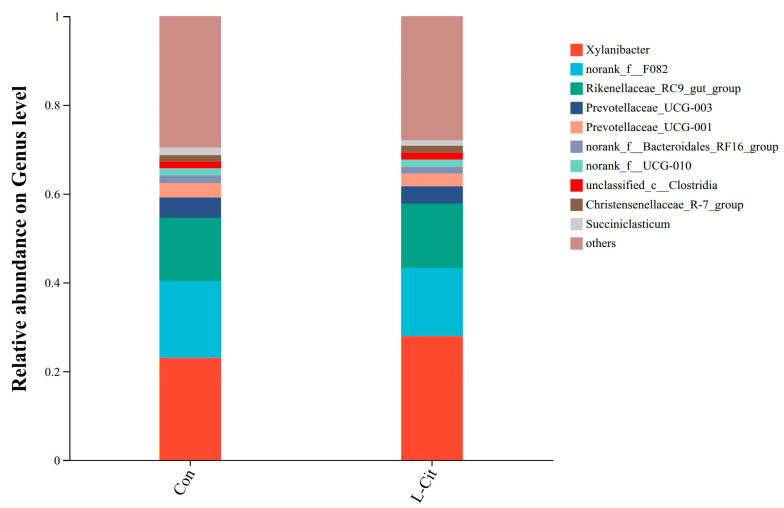
Relative abundance of rumen microbiota at the genus levels in ewes following dietary L-Cit supplementation (n = 6).

As shown in Figure 8, at the genus level, major genera included *Xylanibacter* (23.02%; 27.93%), *norank_f_F082* (17.33%; 15.36%), *Rikenellaceae_RC9_gut_group* (14.22%; 14.50%), *Prevotellaceae_UCG-003* (4.61%; 3.89%), *Prevotellaceae_UCG-001* (3.23%; 2.84%), *norank_f_Bacteroidales_RF16_group* (1.73%; 1.53%), *norank_f_UCG-010* (1.59%; 1.53%), *unclassified_c_Clostridia* (1.51%; 1.58%), *Christensenellaceae_R-7_group* (1.45%; 1.52%), and *Succiniclasticum* (1.68%; 1.24%).

#### 3.3.5. Differential Analysis of Rumen Microbial Species Composition

**Figure 9:**
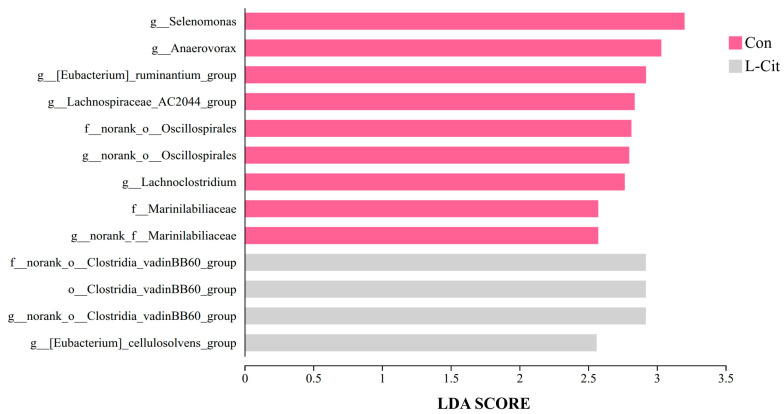
Linear Discriminant Analysis (LDA) of rumen microbiota composition in ewes after dietary L-Cit supplementation (n = 6). Note: The letters o, f, and g denote the taxonomic ranks of phylum, class, order, family, and genus, respectively.

Based on the aforementioned analysis of community structure and species composition, which indicated that dietary L-Cit supplementation induced certain alterations in the rumen microbiota of ewes, we further performed LEfSe (Linear Discriminant Analysis Effect Size) analysis. Using a threshold of LDA score > 2 and a significance level of *p* < 0.05, microbial taxa with significantly differential relative abundance between the two groups were identified. The results of this analysis are presented in Figure 9.

**Figure 10:**
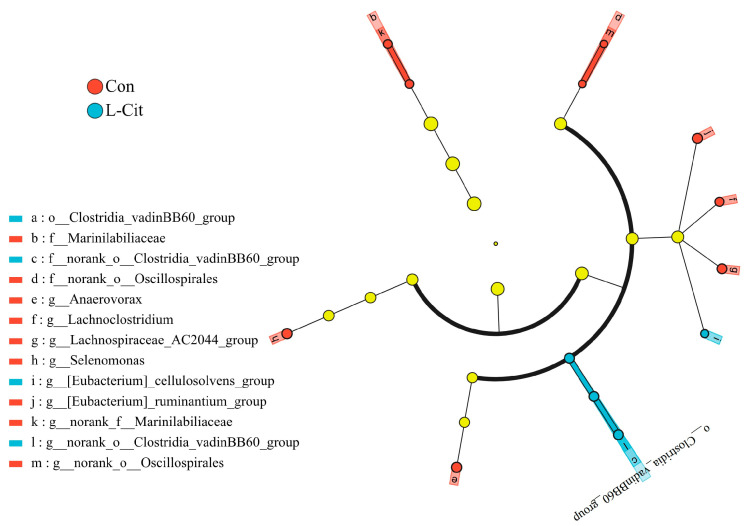
LEfSe (Linear Discriminant Analysis Effect Size) cladogram depicting the taxonomic hierarchy of rumen microbiota composition in ewes after dietary L-Cit supplementation (n = 6). Note: The LDA discriminant bar chart identifies microbial taxa with significant differential abundance among multiple groups, based on Linear Discriminant Analysis.

As shown in Figure 10, at the order level, the relative abundance of *Clostridia_vadinBB60_group* was significantly higher in the L-Cit group (LDA = 2.92, *p* = 0.025). At the family level, relative abundances of *norank_o_Oscillospirales* (LDA = 2.82, *p* = 0.016) and *Marinilabiliaceae* (LDA = 2.57, *p* = 0.045) were significantly higher in the Con group, while the *norank_o_Clostridia_vadinBB60_group* was significantly higher in the L-Cit group (LDA = 2.92, *p* = 0.025).

At the genus level, relative abundances of *Selenomonas* (LDA = 3.20, *p* = 0.025), *Anaerovorax* (LDA = 3.03, *p* = 0.025), *[Eubacterium]_ruminantium_group* (LDA = 2.92, *p* = 0.025), *Lachnospiraceae_AC2044_group* (LDA = 2.84, *p* = 0.025), *norank_o_Oscillospirales* (LDA = 2.80, *p* = 0.016), *Lachnoclostridium* (LDA = 2.77, *p* = 0.006), and *norank_f_Marinilabiliaceae* (LDA = 2.57, *p* = 0.045) were significantly higher in the Con group. In the L-Cit group, relative abundances of the *norank_o_Clostridia_vadinBB60_group* (LDA = 2.92, *p* = 0.025) and the *[Eubacterium]_cellulosolvens_group* (LDA = 2.56, *p* = 0.014) were significantly higher.

#### 3.3.6. Rumen Microbiota Correlation Analysis

**Figure 11:**
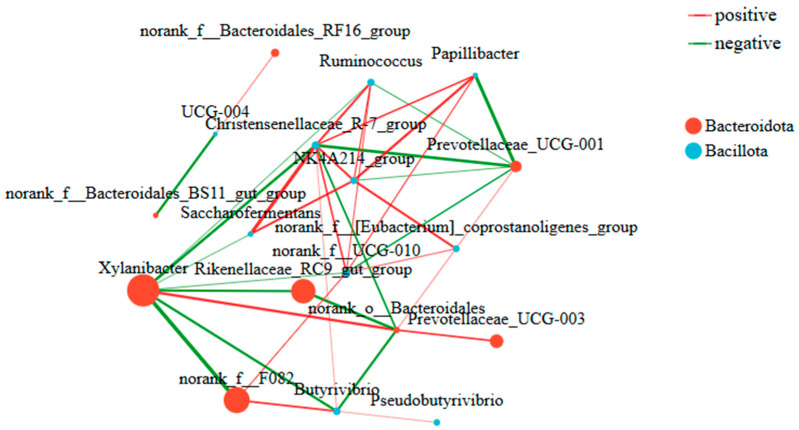
Correlation network analysis of rumen microbiota composition after dietary L-Cit supplementation in ewes (n = 6). Note: Node size represents species abundance. Species belonging to the same higher taxonomic level (e.g., genus) are uniformly colored, with different colors representing distinct higher taxonomic levels. Line color indicates positive (red) or negative (green) correlation. Line thickness corresponds to the magnitude of the correlation coefficient, with thicker lines indicating stronger correlations. A higher number of connecting lines signifies that a species is more closely associated with other species in the network.

As shown in Figure 11, Spearman correlation analysis indicated that *Xylanibacter* was negatively correlated with the *Christensenellaceae_R-7_group*, *Ruminococcus*, *Saccharofermentans*, *norank_f_UCG-010*, *Rikenellaceae_RC9_gut_group*, *Butyrivibrio*, and *norank_f_F082*, and positively correlated with *norank_o_Bacteroidales*. *Rikenellaceae_RC9_gut_group* was negatively correlated with *Xylanibacter* and *norank_o_Bacteroidales*. *norank_f_F082* was negatively correlated with *Xylanibacter* and positively correlated with *norank_f_UCG-010* and *Butyrivibrio*. *UCG-004* was negatively correlated with the *norank_f_Bacteroidales_BS11_gut_group* and positively correlated with the *norank_f_Bacteroidales_RF16_group*.

### 3.4. Correlation Analysis Between Rumen Microbiota and Plasma Reproductive Hormones in Ewes After L-Cit Supplementation

**Figure 12:**
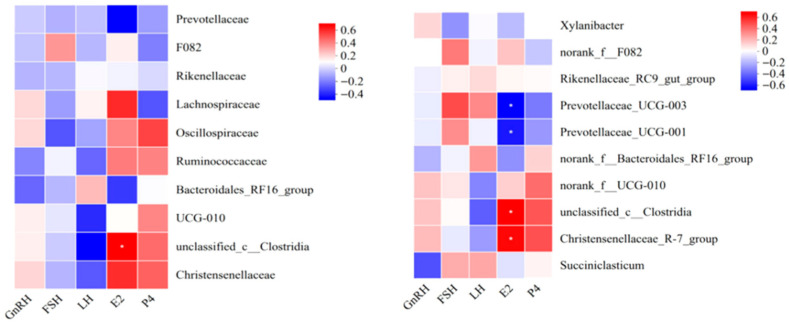
Heatmap of correlation analysis results between rumen microbiota (Family, Genus levels) and plasma reproductive hormones (n = 6). Note: Spearman correlation between relative abundances of top 15 rumen bacterial genera (x-axis) and plasma hormone concentrations (y-axis). R values are displayed in different colors in the figure, and *p* values less than 0.05 are marked with an asterisk (*). The legend on the right shows the color ranges for different R values, where * 0.01 < *p* ≤ 0.05.

As shown in Figure 12, correlation analysis between rumen microbiota and plasma reproductive hormones at the family level revealed that E_2_ content was significantly positively correlated (0.01 < *p* ≤ 0.05) with *unclassified_c_Clostridia* abundance.

As shown in Figure 12, correlation analysis between rumen microbiota and plasma reproductive hormones at the genus level revealed that E_2_ content was significantly positively correlated (0.01 < *p* ≤ 0.05) with *unclassified_c_Clostridia* and *Christensenellaceae_R-7_group* abundances, and significantly negatively correlated (0.01 < *p* ≤ 0.05) with *Prevotellaceae_UCG-003* and *Prevotellaceae_UCG-001* abundances.

## 4. Discussion

### 4.1. Effects of L-Cit Supplementation on Rumen Microbiota in Ewes

A dynamically balanced community structure and relative abundance, which are essential factors that regulate the rumen microbiota in ruminants, play a key role on rumen environmental homeostasis, fiber degradation and nutrient metabolism [16]. Consecutive changes in the rumen ecosystem provide a realistic microbiological explanation on why there was an increased efficiency in the utilization of nitrogen in the initial trial of using L-Cit supplement with ewes [17]. Based on the results of this experiment, it was also found that the alpha diversity indices had no significant differences; this literature suggests that dietary L-Cit supplementation did not produce a significant effect on the total number, and evenness of microbial species in the rumen. This discovery agrees with the idea of rumen microbiome functional redundancy and resilience, where the function of the organization can be modified due to significant diversity changes [18]. The clear separation of the L-Cit and the Con groups in the Principal Coordinates Analysis plot gives credence to the remodeling effect of L-Cit supplementation in the bacterial community. The rise in the dispersion of the L-Cit group could be indicative of increased dynamics and individual difference in rumen microbial response to L-Cit. This inconsistency may show the difference in the ability of individual ruminants to process L-Cit.

On the phylum level, both *Bacteroidota* and *Bacillota* were usual and essential elements of the rumen microbiota that were not affected by the L-Cit supplementation as it was found in other studies [19,20], The results showed that supplementation of L-Cit significantly changed the ruminal nitrogen metabolism and abundance of specific genera in sheep, but the relative abundance of *Bacteroidota* and *Bacillota* at phylum level was not significantly changed, which was consistent with the results of this experiment [7]. *Bacteria* using hemicellulose, including *Xylanibacter*, had a predilection towards an increased range of abundances in the L-Cit-treated sample. LEfSe analysis showed additional changes that were more definite which showed a significant enhancement of the *Clostridia_vadinBB60 group* and *[Eubacterium]_cellulosolvens* group in L-Cit group. *Clostridia_vadinBB60_group* can be generally identified to be anaerobic and complex carbohydrate metabolism responding and *[Eubacterium]_cellulosolvens* is a classic fibrolytic bacterium that can degrade cellulose [21,22,23]. The enhanced richness of the groups implies that L-Cit can result in a more favorable rumen microenvironment that encourages fibrolytic activity. At the same time, the decrease in prevalence of such genera as *Selenomonas* and *Anaerovorax* in the L-Cit group can mean that rumen fermentation changed and no longer serves the proteolytic pathway, or there can be more effective microbial cross-framing processes [24,25].

L-Cit as an antecedent of L-Arg could, when taken up or by direct contact with rumen microbes, have an impact on the rumen pool of L-Arg and polyamines, which act as sources of nitrogen or as growth factors to particular populations of bacteria [7]. Also, conversion of L-Arg to NO can be an important factor, since NO was shown to have selective antimicrobial and signaling functions that can also regulate microbial communities [26]. Indirectly, an increase in fibrolytic bacteria enrichment might be a result of improved host nitrogen metabolism as well as increased availability of nitrogen required to synthesize microbial proteins, thus providing a more reasonable environment to the bacteria [27]. Results of correlation analysis of rumen microbiota indicated that there was a negative relationship between *Xylanibacter* and members of the *F082* family, which highlights the complex co-occurrence network between rumen microbiota. L-Cit dietary supplementation can potentially modify the rumen microbial association network, and thus form a new type of ecological equilibrium, which in turn accumulate better functions of the ecosystem, including fiber digestion [23]. By increasing the ability to digest fibers better, the energy supply of the Turpan black ewes can increase hence saving amino acids used in energy production. This conservation improves the efficacy of synthesizing dietary nitrogen to host tissue and microbial protein [19].

### 4.2. Effects of L-Cit Supplementation on Plasma Reproductive Hormone Levels in Ewes

Effects of L-Cit supplementation on reproductive hormone levels in ewes. The study primarily found that, compared to the Con group, L-Cit supplementation did not significantly alter the concentrations of GnRH, E_2_, P_4_, FSH, or LH in plasma on days 7 and 14 (*p* > 0.05). The modest variation in percentages, though statistically not significant, has shown that L-Cit might not be a strong hypothalamic–pituitary–gonadal (HPG) axis regulator in the present experiment conditions.

This minor, non-significant change in the level of GnRH is in line with the established purpose of L-Cit as a precursor of L-arginine that is used to synthesize the nitric NO [28]. Nitric oxide is also alleged to stimulate GnRH secretion of hypothalamus [29]. However, the fact that this marginal increase occurred, indicates that the dosage or time period that L-Cit was taken was perhaps not enough to produce an intense neuroendocrine signal. This latter negative result of no substantial attenuation in downstream pituitary gonadotropins also confirms the proposition that the weak GnRH stimulus was not adequate to balance out the tonic inhibitory feedback mechanisms controlling the HPG axis [30].

Surprisingly, even though GnRH increased insignificantly, both E_2_ and P_4_ scaled levels exhibited a steady, but insignificant, tendency towards decreasing. L-Cit has been found to affect the steroidogenesis in peripheral tissues. As an example, it has been discovered that NO can regulate the activity of aromatase and steroidogenic enzymes in the granulosa cells [31,32]. L-Cit could have had a small indirect action on the level, by the direct effect of increased production of NO, resulting in a minor diminution of sex steroid production [33]. This peripheral effect may be independent of significant variations in gonadotropins of the pituitary gland which may be the reason why E_2_ and P_4_ decreased but the FSH/LH did not vary significantly compared to the control arm [34].

The dietary intervention was specifically sensitive to the hypothalamic–pituitary axis as the changes in FSH and LH remained to be the lowest possible. This is a property of healthy, reproductively fit subjects in non-stressful states, in which homeostatic processes have a great control over gonadotropin secretion [35,36]. The absence of substantial change may also be because of the physiological condition of the animals used in this trial which was specific or maybe that the control group had an optimal level of basal hormones [37].

Contrary to our hypothesis, L-Cit supplementation did not significantly alter plasma concentrations of estradiol, progesterone, FSH, or LH at day 7 or day 14 compared to control. This indicates that, under the current study conditions, dietary L-Cit did not demonstrably activate the HPG axis or stimulate reproductive hormone synthesis. The numerical differences in oestrus rate are not supported by hormonal evidence.

### 4.3. Impact of L-Cit Supplementation on the Association Between Rumen Microbiota and Reproductive Hormones in Ewes

The correlation analysis data indicates *Fibrobacterota* is one of the disintegrating microorganisms that perform crucial functions in the rumen by decomposing cellulose [38]. Negative relationships among groups of microbes suggest that there is competition or ecological niche divergence within communities that decompose cellulose. These complex correlation networks of rumen microorganisms indicate that the change in microbial communities in one location, brought about by L-Cit, can be cascaded even on the microbial ecosystem across the digestive tract [19]. The exchange of nutrient microbe-microbe interactions or competition usually achieves such interactions because of the exchange of microbial metabolites or the direct interactions between microbes. Ruminants that live on the rumen have clostridia which have been traditionally known to ferment carbohydrates to generate SCFAs [39]. SCFAs, at least acetate, propionate and butyrate, are important sources of energy to ruminants, and have been shown to have an effect on host metabolism and endocrine activity [40]. The positive correlation that was observed between E_2_ and some *Clostridia* bacteria suggests that these microbes can indirectly increase the functioning of the ovaries. This may be by the synthesis of metabolites that promote E_2_ production or action or by modulating the energy levels of the host. A proper energy supply is required in follicular growth and production of steroid hormones [41]. Conversely, the correlation between E_2_ and *Prevotellaceae UCG-003* and *Prevotellaceae UCG-001* is negative, so these groups might suppress the level of E_2_. The most common genus in the rumen is *Prevotella* which is mostly engaged in breaking down non-fibrous food materials and proteins [42]. Some species of *Prevotella* could affect steroid production in the ovary indirectly by producing metabolites that could immobilize this process, or indirectly by altering the host metabolic pathways to influence E_2_ levels. The spirochaetota, which are mostly involved in degradation of cellulose in the rumen of ruminants [43], has a complicated trend of positive and negative associations. This implies that *Spirochaetota* are largely involved in the ecological stability of rumen microbial communities and their changes in abundance can affect the development and activity of other important functional microbes.

While we observed several significant correlations between rumen bacterial genera and hormone concentrations, it is important to emphasize that correlation does not establish causality. Whether changes in rumen microbiota directly influence circulating hormone levels, or whether hormonal variations alter the rumen environment, cannot be determined from our data. Mechanistic studies (e.g., microbiota transplantation or gene knockouts) are required to test causal relationships.

### 4.4. Effects of L-Cit Supplementation on Reproductive Performance of Ewes

**Figure 13:**
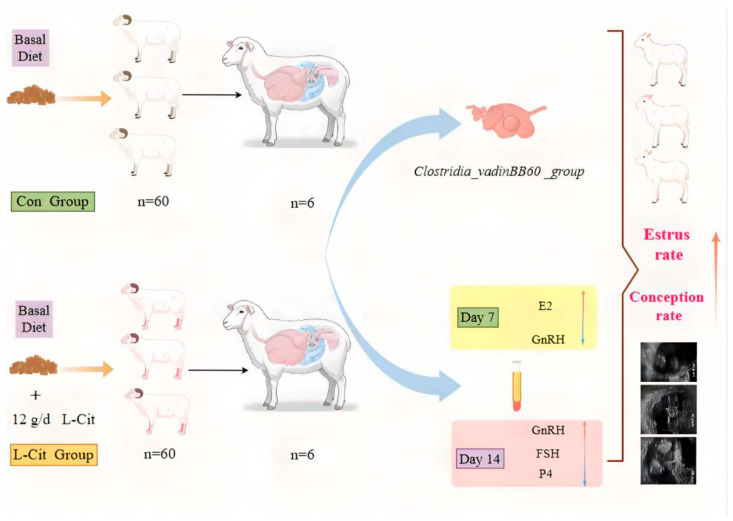
Effects of L-Cit supplementation on rumen microbiota and reproductive performance of ewes.

The critical measures of reproductive performance in sheep are oestrus and conception rate. Exogeneous nutrient supplementation has been found to increase conception and lambing rates in ewes through the inversion of the uterine environment and endometrial status. Characterized studies have shown that arginine is a conditionally necessary amino acid and a dietary supplement, which can be used to boost reproductive performance in sheep and improve uterine environment, thus promoting pregnancy [44]. There is empirical evidence that supplementation with arginine in embryos at an early age has the potential to improve embryo survival rates in sheep. Investigations that were conducted on injecting ewes with arginine have shown that the arginine-treated group had a greater number of oestrus compared to the control group. It was also characterized by significantly enhanced fertility, whereas pregnancy and birth rate were significantly higher than in the control group, and the rate of infertility was also lower. NO produced by means of nitric oxide synthase of arginine, is a cellular relaxing factor and an important signaling molecule in a wide range of reproductive processes, including ovarian follicular growth, ovulation, luteal activity, and uterine receptivity [30]. Studies have shown that NO is critical in the increase in blood circulation to the reproductive organs, which is fundamental in the supply of nutrients and hormones to the developing follicles and the corpus luteum [29]. In addition, L-Cit nutrition has also been said to enhance the uterine circulation and fetal development in sheep [45]. The results of the research reveal that L-Cit supplementation increases oestrus and conception rates in ewes, probably because of better NO production, which consequently does not only improve the functioning of reproductive organs but also affects the reproductive axis on the multi-levels of regulation. The estrus rate in the L-Citrulline group was numerically higher by 15.78% (93.33% vs. 77.55%), but this difference was not statistically significant (*p* = 0.32). This non-significant difference may be attributed to the high variability inherent in ewes’ estrus responses. Therefore, under the current experimental conditions, we cannot conclude that L-Citrulline exerts a genuine biological effect on estrus induction (Figure 13).

## 5. Conclusions

In conclusion, dietary L-Cit supplementation at 12 g/d did not significantly alter plasma reproductive hormone concentrations, oestrus rate, or rumen microbiota composition in ewes under the conditions of this study. Although oestrus rate was numerically higher in the L-Cit group, the difference was not statistically significant, and conception rate was 100% in both groups. Correlation analyses suggested some associations between rumen bacteria and hormone levels, but these do not imply causation. Further research with larger sample sizes and direct mechanistic approaches is warranted to evaluate potential effects of L-Cit in small ruminant reproduction.

## Figures and Tables

**Table 1:** Composition and nutritional levels of the basal diet (dry matter basis) %.

Ingredients	Content	Nutrient Levels	Content
Whole corn silage	35.15	DM	59.26
Corn husk	35.15	CP	12.87
Premix ^(1)^	15.07	EE	3.08
Sorghum stalks	7.54	Ash	5.54
30-peptide	6.21	NDF	26.54
NaHCO3	0.51	ADF	14.03
NaCl	0.31	Ca	0.43
Vitamin D3	0.03	P	0.36
Sodium selenite	0.03	ME (MJ/kg) ^(2)^	10.85

Note: ^(1)^ The premix provided the following per kg of the concentrate supplement. Fe (as ferrous sulfate) 20.5 mg, Zn (as zinc sulfate) 23.2 mg, Cu (as copper sulfate) 5.7 mg, Se (as sodium selenite) 0.7 mg, Ca (as calcium iodate) 1.3 mg, Mg (as magnesium oxide) 0.4 mg, Co (as cobalt chloride) 3.6 mg, VA 6000.0 IU, VD 30.7 mg, VE 8.0 mg. *^(^*^2)^ Metabolic energy is calculated, other nutrient levels were measured values.

**Table 2:** Effects of L-Cit supplementation on ewe reproductive performance (n = 60).

Item	Con Group		L-Cit Group	
	Number	Oestrus Rate %	Number	Oestrus Rate %
Oestrus rate statistics	37/60	61.67	44/60	73.33
X^2^	0.99			
*p*-value	0.32			
Conception rate	37/37	100.00	44/44	100.00
X^2^	—			
*p*-value	—			
Post-trial period (h):				
0	3	5.00 (3/60)	5	8.33 (5/60)
24	4	11.67 (7/60)	3	13.33 (8/60)
48	7	23.33 (14/60)	9	28.33 (17/60)
72	6	33.33 (20/60)	11	46.67 (28/60)
96	8	46.67 (28/60)	7	58.33 (35/60)
120	6	56.67 (34/60)	5	66.67 (40/60)
144	3	61.67 (37/60)	4	73.33 (44/60)

Note: Oestrus rate = (number of oestrus ewes/total ewes) × 100%; conception rate = number of conception ewes/number of breeding ewes × 100%.

## Data Availability

The datasets presented in this study can be found in the NCBI repository under BioProject accession number PRJNA1438583, available at: http://www.ncbi.nlm.nih.gov/bioproject/PRJNA1438583 (accessed on 17 March 2026).

## Abbreviations

The following abbreviations are used in this manuscript:

ADF	Acid Detergent Fiber
BW	Body Weight
CP	Crude Protein
DM	Dry Matter
E_2_	Estradiol
EE	Ether Extract
FSH	Follicle-Stimulating Hormone
GnRH	Gonadotropin-Releasing Hormone
L-Cit	L-Citrulline
LH	Luteinizing Hormone
NO	Nitric Oxide
P_4_	Progesterone
RNA	Ribonucleic Acid
TMR	Total Mixed Ration
